# Magnesium Bioresorbable Scaffold (BRS) Magmaris vs Biodegradable Polymer DES Ultimaster in NSTE-ACS Population—12-Month Clinical Outcome

**DOI:** 10.1155/2022/5223317

**Published:** 2022-12-20

**Authors:** Piotr Rola, Adrian Włodarczak, Szymon Włodarczak, Mateusz Barycki, Marek Szudrowicz, Magdalena Łanocha, Łukasz Furtan, Katarzyna Woźnica, Jan Jakub Kulczycki, Joanna Jaroszewska-Pozorska, Michalina Kędzierska, Adrian Doroszko, Maciej Lesiak

**Affiliations:** ^1^Witelon Collegium State University, 59-220 Legnica, Poland; ^2^Department of Cardiology, Provincial Specialized Hospital in Legnica, 59-220 Legnica, Poland; ^3^Department of Cardiology, The Copper Health Centre (MCZ), 59-300 Lubin, Poland; ^4^Adalbert's Hospital, 61-144 Poznan, Poland; ^5^Faculty of Mathematics and Information Science, Warsaw University of Technology, 00-662 Warsaw, Poland; ^6^Faculty of Medicine, Wroclaw Medical University, 50-556 Wroclaw, Poland; ^7^Clinical Department of Internal and Occupational Diseases, Hypertension and Clinical Oncology, Wroclaw Medical University, 50-556 Wroclaw, Poland; ^8^1st Department of Cardiology, Poznan University of Medical Sciences, 61-491 Poznan, Poland

## Abstract

**Background:**

Percutaneous coronary intervention (PCI) in the acute coronary syndrome (ACS) setting is associated with a greater probability of device failure. The currently ongoing development of new scaffold technologies has concentrated an effort on improving the PCI outcomes, including the use of new biodegradable materials. This pilot study evaluates the performance of a magnesium bioresorbable scaffold (Magmaris, Biotronik, Germany) in comparison to the sirolimus‐eluting bioresorbable polymer stents (BP-SES) (Ultimaster, Terumo, Japan) in the NSTE-ACS setting.

**Methods:**

The population of this pilot comprised 362 patients assigned to one of two arms (193-Magmaris vs 169-Ultimaster). The data regarding the primary outcome comprised of death from cardiac causes, myocardial infarction, and stent thrombosis, along with target-lesion failure (TLF) and other clinical events was collected in the 1-yearfollow-up.

**Results:**

There were no statistically significant differences in clinical outcomes in the short term (30 days) or in the 1-yearfollow-up between both groups.

**Conclusion:**

At 12 months, there were no statistically significant differences between the Magmaris and Ultimaster for composed endpoints or the TLF.

## 1. Introduction

The clinical outcomes of patients with coronary artery disease (CAD) undergoing percutaneous coronary intervention (PCI) have remarkably improved following the introduction of second-generationdrug-eluting stents (DES). Nevertheless, PCI in an acute coronary syndrome (ACS) setting remains associated with an increased rate of device failure [1]. These phenomena might be connected with the exacerbation of local inflammatory response to the metallic scaffold as well as the prevalence of permanent polymer coatings of a scaffold bone [2]. Recently, several biodegradable materials have been proposed in order to overcome these limitations.

One of the concepts assumed replacing of permanent polymer with a biodegradable substitute, which allowed for gradual drug release and leave bare metal scaffold in place facilitating local reendothelialization, reducing thus the inflammatory reaction and in consequence lowering the rate of late stent-related complications [2].

The other strategy is related to the complete resorption of scaffolds (BRS) and allows for anatomical and functional restoration of the vessel without maintaining any material in the treated vessel in the long-term outcome.

The first generation of BRS (Absorb) was built from biodegradable poly-L-lactide (PLLA) and had initial encouraging outcomes [3]. However, longer clinical observation has raised safety-related concerns. The data from registries suggested an increased rate of scaffold thrombosis and target vessel myocardial infarction. Therefore, after publishing the results of Absorb II [4] and Absorb III [5] trials, the Absorbs have been withdrawn from commercial use in clinical practice.

Despite the initial setback of the BRS technology, a new generation of bioresorbable scaffolds has recently appeared. The Magmaris is a novel scaffold with a backbone made of absorbable magnesium alloy—fully coated with biodegradable PLLA polymer BIOlute. The initial data suggested a reasonable safety profile of this device. Nevertheless, the data regarding the head-to-head comparison between Magmaris and the novel generations of DES is still sparse [6].

Hence, this pilot study was designed to investigate the performance of a magnesium bioresorbable scaffold (Magmaris) in comparison to the sirolimus‐eluting bioresorbable polymer stents (BP-SES) Ultimaster in subjects with the non-ST elevation acute coronary syndrome (NSTE-ACS).

## 2. Materials and Methods

### 2.1. Study Population

**Figure 1:**
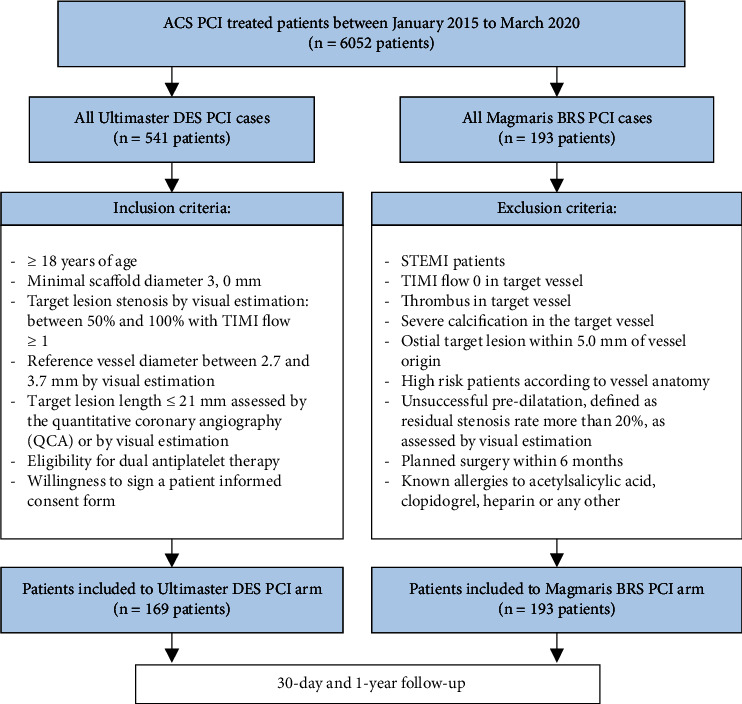
Study flow diagram with inclusion and exclusion criteria.

The study was based on a retrospective two-center analysis of two NSTE-ACS-Registries conducted at the Clinical Departments of Cardiology. The study population comprised 362 patients who were assigned to one out of two study arms. The first one consisted of 193 patients treated with Magmaris implantation. The second arm was composed of 169 subjects treated with Ultimaster implantation. All participants were diagnosed with acute coronary syndrome according to current guidelines (with the exclusion of ST-segment elevation myocardial infarction (STEMI) cases) and had a clinical indication for PCI. All the inclusion and exclusion criteria are pooled in Figure 1.

Out of all NSTE-ACS-Ultimaster cases (541) performed between January 2015 and December 2018 at our Cardiac departments, we carefully selected 169 patients meeting the inclusion and exclusion criteria. The BRS group was formed by the 193 NSTE-ACS subjects.

### 2.2. PCI Procedures

**Figure 2:**
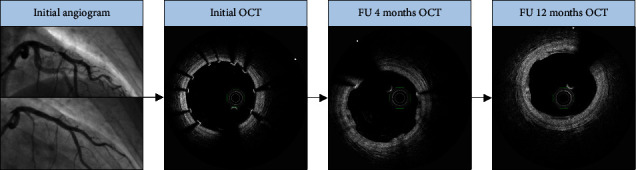
Magmaris implantation procedure with subsequent OCT follow-up.

All the PCI procedures were initiated with a predilation (using a noncompliant (NC) balloon, sized with a 1 : 1 balloon to artery ratio) and followed by mandatory postdilatation with an NC balloon (at least 16 atm) sized 1 : 1 balloon/scaffold ratio or higher. A decision regarding the use of IVUS/OCT support during the PCI was left to the discretion of the operators. An example of the BRS implantation procedure with subsequent follow-up with additional evaluation in intravascular imaging is presented in Figure 2.

### 2.3. Study Devices

The Magmaris, initially known as a DREAMS 2G, is a metallic magnesium sirolimus-eluting scaffold covered with a biodegradable polymer (BIOlute) Poly-L-Lactide (PLLA) and is available in two diameter sizes (3.0 mm and 3.5 mm). The approximate scaffold bioresorption time is 12 months.

The second device used in this study was Ultimaster–an ultrathin cobalt-chromiumsirolimus-eluting stent, coated with a biodegradable poly-(D, L-lactide-co-caprolactone) copolymer (PDLLA-PCL) with an average degradation time of 3-4 months.

### 2.4. Outcomes and Endpoints

The primary outcomes included

- death from cardiac causes
- myocardial infarction
- in-stent thrombosis

The secondary outcomes were device-orientated and defined as follows:

- target-lesion failure (TLF) including death
- target vessel-related myocardial infarction (TV-MI)
- target lesion revascularization (TLR)

Other clinical variables were collected, including scaffold restenosis, death from any reason, cerebrovascular episodes, need for any revascularization procedure, or myocardial infarction.

All the clinical data were obtained by trained staff (physicians or/and nurses) during personal visits or telephone contact during the 30-day and 1-yearfollow-up period. The definition of myocardial infarction was based on the Fourth Universal Definition of Myocardial Infarction [7].

### 2.5. Statistical Analysis

The R language was used for analyses. Continuous variables were characterized by their mean and standard deviation, or median and interquartile range, dependent on their distribution, whereas the frequencies were used for categorical variables. The study subjects were compared between groups using a two-sample Mann–Whitney's or *T*-Test as an appropriate test for continuous variables and Fisher's Exact Test for categorical variables. The Bonferroni correction was applied to adjust for the multiple comparisons. The power calculation was performed using the power.fisher.test from a statmod R package. *p* value <0.05 was considered statistically significant.

## 3. Results and Discussion

### 3.1. Patient Characteristics

All the data regarding patients' clinical characteristics are presented in Table 1. In both study arms majority of subjects were male (77.7% in the Magmaris and 75.7% in the Ultimaster arm, respectively). The primary diagnosis of NSTEMI was more common in the Magmaris than in the Ultimaster group (84.5% vs 52.1%, respectively, *p* < 0.001) Among all comorbidities atrial fibrillation was less frequently observed in the Magmaris group (4.6% vs 14.2%, respectively, *p* = 0.002). Similarly, lower total cholesterol (4.6 ± 1.3 vs 4.95 ± 1.4 mM; respectively, *p* = 0.041) and LDL cholesterol levels were observed in the Magmaris group (2.5 ± 1.2 vs 2.92 ± 1.9 mM respectively, *p* = 0.025). Additionally, subjects from the Magmaris arm were characterized by higher left ventricular ejection fraction at discharge time (60.4% ± 10.9 vs 53.6% ± 13.1 respectively, *p* = 0.001). The average duration of hospitalization was shorter in the Magmaris group (2.7 ± 1.8 vs 3.9 ± 2.9, respectively)

### 3.2. PCI Characteristics

Subjects from the Magmaris arm maintained slightly more aggressive lesion preparation (mean pressure of 17.7 ± 0.8 vs 15.9 ± 1.9 atm., respectively, *p* < 0.001). Noteworthy, there were no differences in the size of the balloon catheter used for predilatation in both arms. Similar differences were observed regarding the postdilatation parameters size (0.25 mm greater than scaffold-65.2% vs 20.7%, respectively, *p* < 0.001; and 0.5 mm greater than scaffold 18.2% vs 8.3%, respectively, *p* < 0.001), as well as regarding the mean pressure used during postdilatation (17.7 ± 0.8 for Magmaris vs 16.7 ± 1.0 atm. for Ultimaster respectively, *p* < 0.001). On the other hand, significantly lower radiation was used during the Magmaris implantation (1056.7 ± 697.8 vs 1244,2 ± 761.1 mGy respectively, *p* = 0.008). All data regarding procedural characteristics were collected in Table 2.

### 3.3. Clinical Outcomes

All the data regarding clinical outcomes are summarized in Table 3. Except for the 30-dayfollow-up “any other revascularization” rate (0% vs 5%, respectively, *p* = 0.012) there were no differences in clinical outcomes in the 30 days follow-up between both study arms. At 1-yearfollow-up, no significant differences among primary and secondary endpoints between both study groups were noticed. However, in the Magmaris cohort, a slightly lower rate of the primary endpoint (1.5% vs 5% respectively, *p* = 0.074) which however did not reach statistical significance in this pilot study. A similar trend was observed for the principal secondary outcomes (1.5% vs 5.4%, respectively, *p* = 0.199). Moreover, in the Magmaris arm, we reported a lower rate of other coronary revascularization (9.3% vs 14% in the Ultimaster arm, respectively, *p* = 0.188) again without statistical significance. The power calculations revealed that for comparing 3 outcomes out of 193 vs. 9 outcomes of 169, the power of the Fisher test at 0.05 significance level was 0.467.

## 4. Discussion

This is the first report comparing the 1-year clinical outcome of two sirolimus‐eluting magnesium bioresorbable scaffolds (Magmaris), and ultrathin cobalt-chromium stent coated with a bioresorbable polymer (Ultimaster) in NSTE-ACS conditions.

Since publishing the data from the first-in-man trial [8] and receiving the CE mark, the Magmaris scaffold has been proven to be a relatively safe device. However, evidence coming from small-size preclinical studies [9, 10], along with initial clinical trials -mainly focused on patients with stable angina [11] and acute coronary syndrome [12] supported this statement. Although recently published midterm and long-term outcomes [13–18] are also encouraging, the data from trials comparing Magmaris with the new DES generation are still scarce. As result, we performed this pilot study to evaluate the utility of a magnesium bioresorbable scaffold (Magmaris) in comparison with an ultrathin cobalt-chromium stent (Ultimaster) in the acute coronary syndrome setting.

The main findings of this study are as follows:

1. In our relatively small, retrospective, nonrandomized study cohort Magmaris and Ultimaster showed no differences in clinical outcomes for the Primary Endpoint (death from cardiac causes, myocardial infarction, in-stent thrombosis) as well as regarding the TLF in the 30-day and 1-year-follow-up period
2. Magmaris did not present any definite scaffold-related thrombosis after a 12-month observation period

In recently conducted studies Ultimaster has demonstrated good overall device performance, in the ACS cohort [19]. These favorable ACS-related outcomes might be linked to the presence of biodegradable polymer, which intensifies the alleviation of vascular inflammation and accelerates endothelial maturation, which is crucial in these high-risk patients [20]. Considering that in our study the rates of 1-year composite endpoints-TLR in the Ultimaster arm were lower than previously described [21] (5.4% *vs* 7.9%), we might assume that the Magmaris might demonstrate the outcomes compared to the ones observed in the new-generation DES devices.

A similar suggestion was made by Hideo–Kajita et al. [6], wherein the subpopulation of non-ACS subjects, no significant differences between the Magmaris and other BP-SES (Orsiro) were observed, but again was higher than observed in our study (6% for Magmaris *vs* 6.4% in Orsiro group). On the other hand, a small study by Toušek et al. [22] suggests that both QCA and OCT revealed lower efficiency of Magmaris scaffold when compared to the leading drug-eluting metallic stent (Xience) in the 12-monthfollow-up period of patients with STEMI. However, a detailed analysis of the implantation technique used in this study reveals deviations from the recommended BRS Magmaris “4P strategy” [23], particularly in terms of lesion preparation.

These results were partially confirmed in MAGSTEMI randomized control trial [24] which evaluated the 1-year outcome of STEMI patients treated with implantation of Magmaris or ultrathin, biodegradable polymer sirolimus-eluting stent (Orsiro). The rate of the device-oriented endpoint (TLR) was significantly higher in the Magmaris group (16.2% *vs* 5.3%), despite the use of a dedicated implantation technique [25]. However, patients after Magmaris implantation showed enhanced in-deviceendothelium-independent and endothelium-dependent vasomotor response, compared to the Orsiro subpopulation. Also, like in our study, no thrombotic safety concerns occurred, despite the highly thrombogenic setting. We can partly attribute a relatively low rate of thrombosis to scaffold backbone features-higher radial strength which reduces time-dependent recoil phenomena and improves the local hemodynamic properties affecting the endothelization period [9, 26, 27]. In addition, magnesium used to scaffold production has got favorable electrochemical properties -compare to other metals used for implants is more electronegative and may repel negatively charged platelets leading to the indirect reduction of potential thrombogenicity [27, 28].

The selection process for our study was based on the inclusion and exclusion criteria of the Magmaris registry. We qualified patients with rather “unchallenging” anatomy of the lesions without complex calcified cases, requiring implantation of multiple scaffolds (including two stent techniques for bifurcation lesions). This could constitute some limitations of our study, however, what needs to be emphasized, the aim of this pilot study was focused on the clinical evaluation of Magmaris—a novel BRS that has not been widely used in clinical practice (implanted mainly in selected cardiac centers familiar with the magnesium BRS technology). Therefore, we believe that the results of our study despite some limitations may provide valuable information regarding the safety and efficacy of the magnesium BRS in the real-life NSTE-ACS setting.

## 5. Limitations

This study has several limitations. First, the data were collected retrospectively in the relatively short-term observation period (1-year follow-up). Second, this is a comparison between the 2 non-randomized observational registries. Third, performed power calculation revealed that in terms of the 30-day and 1-yearfollow-up, the study is underpowered, which might be however justified by its pilot character. Fourth, the study groups were not fully homogenous several differences among clinical features were observed. Finally, even though we can presume that the low rate of image-guided PCI performed in this study could have affected the outcomes, the study population consisted of NSTE-ACS patients, where the use of the OCT/IVUS procedures in an emergency setting is less frequent, which makes this study more relevant to the real-life clinical scenario.

## 6. Conclusions

In both study arms (BRS and Ultimaster), no definite scaffold-related thrombosis occurred after 12 months of follow-up. We have observed similar outcomes in terms of the two major composed endpoints—primary outcome (including death from cardiac causes, myocardial infarction, and in-stent thrombosis) and TLF. Nevertheless, the pilot character of this study and relatively low sample size point to the need for a subsequent large multicenter prospective trial in order to address precisely the efficacy and safety concerns of BRS, as well as to draw final conclusions and formulate precise recommendations in this matter.

## Figures and Tables

**Table 1:** Study patient characteristics.

	Magmaris patients *N*-193	Ultimaster patients *N*-169	*p* value
Age	66.3 ± 8.9	65.2 ± 9.34	0.605
Gender-male (ratio)	150 (77.7%)	128 (75.7%)	0.481
Unstable angina	30 (15.5%)	81 (47.9%)	<0.001
NSTEMI	163 (84.5%)	88 (52.1%)	<0.001
Diabetes mellitus type 2	72 (37.3%)	59 (34.9%)	0.789
Oral antidiabetic treatment	58 (30%)	45 (26.6%)	0.724
Insulin	14 (7.2%)	14 (8.2%)	0.844
Hypertension	171 (88.6%)	158 (93.5%)	0.143
Hyperlipidemia	152 (78.7%)	130 (76.9%)	0.812
Atrial fibrillation	9 (4.6%)	24 (14.2%)	0.002
Previous PCI	78 (40.4%)	61 (36.1%)	0.443
Primary diagnosis of MI	59 (30.5%)	60 (35.5%)	0.499
LV-EF	60.4% ± 10.9	53.6% ± 13.1	<0.001
Total cholesterol (mmol/L)	4.6 ± 1.3	4.95 ± 1.4	0.041
LDL (mmol/L)	2.5 ± 1.2	2.92 ± 1.9	0.025
Triglycerides (mmol/L)	1.8 ± 1.8	1.6 ± 0.8	0.181
Creatinine (µmol/l)	84.1 ± 22.2	82.9 ± 21.9	0.767
Days of hospitalization	2.7 ± 1.8	3.9 ± 2.9	0.041

Abbreviations: NSTEMI, non-ST elevation myocardial infarction; TIA, transient ischemic attack; PCI, percutaneous coronary intervention; LV-EF, left ventricular ejection fraction; MI, myocardial infarction.

**Table 2:** Procedural characteristic.

Procedural characteristic	Magmaris patients *N*-193	Ultimaster patients *N*-169	*p* value
Treated vessel: LAD	80 (41.4%)	65 (38.4%)	0.592
LCX	49 (25.3%)	47 (27.8%)	0.634
RCA	61 (31.6%)	56 (33.1%)	0.626
IM	3 (1.6%)	1 (0.6%)	0.822
Predilationballoon: mean diameter (mm)	3.2 ± 0.3	3.1 ± 0.3	0.092
Mean pressure (atm.)	17.7 ± 0.8	15.9 ± 1.9	<0.001
Average scaffold number	1.1 ± 0.2	1.2 ± 0.4	0.482
Average scaffold diameter (mm)	3.28 ± 0.27	3.24 ± 0.31	0.035
Average scaffold length (mm)	20.8 ± 3.3	23.9 ± 4.1	0.041
Postdilationballoon: mean diameter (mm)	3.5 ± 0.3	3.3 ± 0.3	<0.001
Mean pressure (atm.)	17.7 ± 0.8	16.7 ± 1.0	<0.001
0.0 mm greater than the scaffold	31 (16.6%)	120 (71%)	<0.001
0.25 mm greater than the scaffold	130 (65.2%)	35 (20.7%)	<0.001
0.5 mm greater than the scaffold	32 (18.2%)	14 (8.3%)	<0.001
Contrast agent volume (ml)	151.5 ± 65.4	148.5 ± 68.5	0.419
Dose of radiation (mGy)	1056.7 ± 697.8	1244.2 ± 761.1	0.008
OCT/IVUS guided PCI	41 (21.2%)	28 (16.7%)	0.521
Number of edge dissections	7 (3.6%)	7 (5%)	0.894
Perforation of the vessel	0 (0%)	0 (0%)	1
Side branch occlusion	2 (1%)	1 (0.6%)	1
Antiplatelet therapy acetylsalicylic acid	191 (98.9%)	167 (98.8%)	1
Clopidogrel	76 (38.9%)	148 (87.5%)	<0.001
Ticagrelor	117 (60.6%)	20 (11.9%)	<0.001

Abbreviations: LAD–left anterior descending artery; Cx–circumflex artery; IM-intermedium artery; RCA-right coronary artery; OCT-optical coherent tomography; IVUS-intravascular ultrasound.

**Table 3:** Clinical outcomes in both study arms.

Clinical outcomes	Magmaris patients *N*-193	Ultimaster patients *N*-169	*p* value
30-day FU primary outcome (cardiac death, myocardial infarction, in-stent thrombosis)	0 (0%)	2 (1%)	0.217
30-day FU principal secondary outcome target lesion failure (cardiac death, target vessel myocardial infarction, and need for target lesion revascularization)	0 (0%)	0 (0%)	1
30-day FU death			
Any	0 (0%)	0 (0%)	1
Cardiac	0 (0%)	0 (0%)	1
30-day FU myocardial infarction:			
Any other	0 (0%)	2 (1%)	0.217
Target vessel	0 (0%)	0 (0%)	1
30–day FU scaffold:			
Thrombosis	0 (0%)	0 (0%)	1
Restenosis	0 (0%)	0 (0%)	1
30–day FU revascularisation:			
Target lesion	0 (0%)	0 (0%)	1
Target vessel	0 (0%)	0 (0%)	1
Any other	0 (0%)	9 (5%)	0.012
1-year FU primary outcome (cardiac death, myocardial infarction, and in-stent thrombosis)	3 (1.5%)	9 (5%)	0.074
1-year FU principal secondary outcome target lesion failure (cardiac death, target vessel myocardial infarction, and target lesion revascularization)	3 (1.5%)	7 (5.4%)	0.199
1-year FU death:			
Any	2 (1.0%)	0 (0%)	0.501
Cardiac	0 (0%)	0 (0%)	1
1-year FU myocardial infarction:			
Any other	3 (1.5%)	4 (2%)	0.710
Target vessel	2 (1.0%)	5 (3%)	0.259
1-year FU scaffold			
Thrombosis	0 (0%)	0 (0%)	1
Restenosis	2 (1.0%)	2 (1%)	1
1-year FU revascularisation:			
Target lesion	2 (1.0%)	3 (2%)	0.668
Target vessel	3 (1.5%)	7 (4%)	0.199
Any other	18 (9.3%)	24 (14%)	0.188

Abbreviations: TIA, transient ischemic attack; PCI, percutaneous coronary intervention; FU-follow up.

## Data Availability

Data are not included in the manuscript due to local laws and privacy restrictions. It can be made available from the corresponding author upon request.
